# Sulforaphane Ameliorates High-Glucose-Induced Damage in a Diabetic Foot Ulcer Cell Model by Activating the Nrf2 Pathway to Improve Mitochondrial Function and Suppress Inflammation

**DOI:** 10.3390/biomedicines14050997

**Published:** 2026-04-27

**Authors:** Xiao Chen, Zhimin Yin, Rui Jiao, Hui Han, Liangcai Yuan, Jie Zhou

**Affiliations:** Department of Plastic and Burns Surgery, Northern Jiangsu People’s Hospital, Yangzhou University, Yangzhou 225009, China; c18051060600@126.com (X.C.); yinzhimin120044@126.com (Z.Y.); doc_jerry@163.com (R.J.); drgray@126.com (H.H.)

**Keywords:** diabetic foot ulcers, sulforaphane, Nrf2, mitochondrial function, inflammatory

## Abstract

**Background/Objectives**: Diabetic foot ulcers (DFUs) are a common and challenging complication of diabetes, significantly impacting the quality of life for patients due to impaired wound healing. Exploring effective and targeted therapies for DFUs is therefore both important and meaningful. Sulforaphane (SFN), a natural bioactive compound found in cruciferous vegetables, shows promise in this area. However, its role and underlying mechanisms in promoting wound healing in DFUs have not been fully understood. **Methods**: Human umbilical vein endothelial cells (HUVECs) were cultured under high-glucose conditions to establish an in vitro diabetic model. Cell viability, inflammation, apoptosis, and mitochondrial function were assessed. The expression and activation of Nrf2 were examined following SFN treatment. Additionally, Nrf2 overexpression was performed to validate its role in mediating the protective effects of SFN under high-glucose stress. **Results**: High-glucose conditions significantly reduced HUVEC viability and increased inflammation, apoptosis, and mitochondrial dysfunction. Treatment with SFN effectively counteracted these detrimental effects. SFN robustly activated Nrf2 signaling, and overexpression of Nrf2 recapitulated the protective effects of SFN, attenuating cellular damage under high-glucose conditions. **Conclusions**: SFN activates Nrf2 expression and protects HUVECs from high-glucose-induced injury by improving cell viability, mitochondrial function, and inflammatory response. These findings suggest that SFN may serve as a promising targeted therapy for diabetic foot ulcers.

## 1. Introduction

Diabetes mellitus (DM) is a chronic metabolic condition characterized by long-term elevation of blood sugar beyond the normal range [1]. A critical consequence of this condition is the gradual, cumulative damage it inflicts on multiple organ systems, notably the nerves, eyes, heart, kidneys, and vasculature, ultimately precipitating a series of severe complications [2]. Among these complications, foot ulcers caused by peripheral capillary damage, clinically called diabetic foot ulcers (DFU), are one of the most common complications in diabetes patients, bringing long-term pain and suffering to millions of patients worldwide [3]. This condition causes lasting pain and suffering for millions worldwide, with severe cases often resulting in amputation or death [4]. It has been reported that local neuropathy, peripheral vascular occlusion, dysregulation of local cytokine secretion, increased reactive oxygen species (ROS) production, deregulated apoptosis, impaired wound healing, and angiogenesis contribute to DFU development and progression [5]. Among these factors, oxidative stress in local tissues and impaired angiogenesis mechanism are the hot points in the formation and development of DFUs in previous studies, and how to reduce the oxidative stress damage in local tissues and promote the regeneration of blood vessels near the DFU has also become an important topic in the clinical treatment of DFUs [6]. In this regard, scholars have conducted a lot of studies on rat diabetes models, and the results show that reducing oxidative stress [1] and promoting blood duct formation [7] can effectively improve the wound healing ability of DFUs. Surgical debridement, offloading to reduce pressure on the ulcer, and management of lower extremity ischemia and foot infections constitute the primary therapeutic approaches for diabetic foot ulcers [4]. Research indicates that the pathogenesis is due to the imbalance between the microbiota and the immune system, which eventually leads to delayed wound healing [8]. Despite progress in managing diabetic foot ulcers (DFUs) and a better understanding of their healing mechanisms, the underlying molecular processes are still unclear [9].

VEGF is a crucial endogenous growth factor renowned for its role in regulating wound healing [10]. A growing body of evidence from both in vivo and in vitro studies demonstrates that elevated glucose levels contribute to the development of chronic inflammation and local inflammation [11], leading to increased expression of various pro-inflammatory cytokines, including interleukin (IL)-6 [12] and IL-8 [13]. Furthermore, under hyperglycemic conditions, activated endothelial cells produce pro-inflammatory cytokines through local autocrine and paracrine signaling pathways, thereby perpetuating a self-sustaining inflammatory cycle [14]. In addition, high glucose levels enhance the surface expression of adhesion molecules on endothelial cells—such as monocyte chemoattractant protein-1 (MCP-1) and intercellular adhesion molecule-1 (ICAM-1)—which facilitate monocyte recruitment and interaction with inflammatory mediators, thereby sustaining chronic inflammatory states and promoting endothelial dysfunction [15]. It can be seen that finding targets and corresponding drugs to reduce oxidative stress and promote angiogenesis will greatly benefit the clinical treatment of DFUs.

Sulforaphane (SFN), a potent natural activator of the Nrf2 pathway, is widely recognized for its antioxidant properties. In addition, it possesses a broad spectrum of biological activities, including anti-inflammatory, anticancer, epigenetic regulatory, and antibacterial effects [16], and has been implicated in the prevention and treatment of cancer [17], nervous system disorders [18], and coronavirus disease [19]. Consumption of cruciferous vegetables rich in sulforaphane (SFN) has been associated with the prevention of various chronic diseases, including cardiovascular diseases, neurodegenerative disorders [20], and diabetes [21]. Many studies have shown that SFN could activate the AKT and Keap1/2 signaling pathways to alleviate reactive oxidative stress [22,23], and activate the IKB and NFKB signaling pathways to alleviate inflammation [24,25], thereby promoting mouse granulosa cell proliferation via the NRF2–TKT pathway [16], alleviating psoriasis by enhancing antioxidant defense through KEAP1-NRF2 pathway activation, attenuating inflammatory signaling [26], and mediating the function of mitochondrial function to alleviate apoptosis [27]. In diabetic mice, SFN can alleviate the inflammatory response at the wound site by enhancing the phagocytic function of damaged macrophages and promoting the polarization of M2-type macrophages [28]. However, whether this compound can be used as a therapeutic agent for wound healing in patients and models of DFUs, as well as its possible mechanism, requires further investigation. Nrf2 is an important activator of SFN [29,30], as an endogenous transcription factor that regulates antioxidant enzymes, while NF-κB overexpression promotes pro-inflammatory and pro-oxidant states [31]. The role of NRF2 has evolved from being initially recognized as a transcription factor primarily involved in redox homeostasis and detoxification to being widely acknowledged as a central regulator of cellular protein homeostasis, metabolism, and iron regulation [32]. And there are reports stating that Nrf2 could increase intracellular peroxides by upregulating the cystine transporter xCT encoded by Slc7a11, thereby regulating mitochondrial function [33]. The Nrf-2/GPX-4/xCT axis could regulate mitochondrial autophagy and mitochondrial generation in tumor cells [34]. Studies have shown that Nrf2 in serum can serve as a biomarker for screening diabetic foot ulcers and as a potential therapeutic target [35]. Overexpression of SIRT6 enhanced the activation of the Nrf2 pathway in the wound tissues of DFU rats and vascular endothelial cells exposed to HG, thereby reducing the wound area, accelerating epithelial formation, increasing collagen deposition, and improving angiogenesis [36]. Whether SFN can mediate cell function regulation through Nrf2 to counteract high-glucose-induced cell damage remains to be investigated.

In this study, we utilized high-glucose-cultured HUVECs to simulate a diabetic milieu and meticulously examined the influence of SFN on oxidative stress, inflammation, and apoptosis under DFU conditions. Our research reveals that SFN, as a promising compound and excipient, holds potential in facilitating wound healing for patients and models of diabetic foot ulcers (DFUs) by activating the expression of Nrf2. This not only offers a novel perspective on understanding DFUs but also serves as an innovative strategy for their clinical management.

## 2. Materials and Methods

### 2.1. Cell Culture

Human umbilical vein endothelial cells (HUVECs) were procured from the Shanghai Institute of Biochemistry and cultured in Dulbecco’s Modified Eagle Medium (DMEM) supplemented with 10% fetal bovine serum (FBS) (Gibco, Thermo Fischer Scientific, Waltham, MA, USA). The culture conditions were maintained at 37 °C in a humidified incubator under an atmosphere of 5% CO_2_. To emulate the diabetic milieu in vitro, HUVECs were subjected to either normal-glucose conditions (5.5 mM) or high-glucose conditions (30 mM) in DMEM [37,38]. To eliminate the non-specific effects caused by high osmotic pressure, mannitol (24.5 mmol/L) was added to the NG (5.5 mmol/L) solution to make its osmotic pressure consistent with that of the HG (30 mmol/L D-glucose) group.

Sulforaphane (HY-13755) was obtained from MedChemExpress (Monmouth Junction, NJ, USA) and was meticulously prepared by initially dissolving it in DMSO to achieve a concentration of 1 M. Subsequently, this solution was diluted with culture medium to yield a series of precise final concentrations: 0.1, 1.0, 2.0, 6.0, and 10 µM. Notably, the ultimate concentration of DMSO within the culture medium was scrupulously maintained at less than 0.01%. The NG group was treated with the same amount of DMSO.

### 2.2. Assessment of Cell Viability

Cell viability was evaluated using the Cell Counting Kit-8 (Yeasen Biotechnology Co., Ltd., Shanghai, China), following the manufacturer’s instructions with meticulous precision. The optical density was quantified at a wavelength of 450 nm utilizing the advanced Tecan Infinite 200 microplate reader (Sunrise, Tecan, Männedorf, Switzerland). Notably, all experiments were conducted in triplicate to ensure the reliability and reproducibility of the results.

### 2.3. Enzyme-Linked Immunosorbent Assay

The cell supernatants from different groups were carefully harvested and subsequently subjected to centrifugation at 300 g under a controlled temperature of 4 °C for 10 min, ensuring the efficient removal of any sediment. Following this step, the concentrations of IL-6 (70-EK106/2-96), IL-8 (70-EK108-96), ICAM-1 (70-EK189-96), and MCP-1 (70-EK187-96) in the culture media were meticulously quantified and analyzed in accordance with the detailed instructions provided in the enzyme-linked immunosorbent assay (ELISA) kit manual (Multi Sciences, Hangzhou, China).

### 2.4. Analysis of Apoptosis Using Flow Cytometry

The cells from distinct treatment groups were harvested and assessed for apoptosis using the Annexin V-FITC/PI Apoptosis Detection Kit (DCFH-DA, Solarbio, Beijing, China). Specifically, the collected cells were gently washed with ice-cold phosphate-buffered saline, sequentially stained with Annexin V-FITC and PI, and subsequently analyzed by flow cytometry. The resulting data were meticulously evaluated using CytExpert software (Beckman Coulter, Brea, CA, USA). Each measurement was conducted in triplicate, ensuring the analysis of no fewer than 10,000 cells per sample.

### 2.5. Measurement of Reactive Oxygen Species (ROS) Using Flow Cytometry

Reactive oxygen species (ROS) levels in HUVECs were quantitatively assessed via flow cytometry, utilizing ROS Assay Kit (Beyotime, Nantong, China). Following distinct treatments, the cells were meticulously harvested, gently washed with pre-chilled phosphate-buffered saline (PBS), and subsequently incubated with dichlorodihydrofluorescein diacetate (DCFH-DA) in culture medium at 37 °C for 30 min under controlled conditions. Thereafter, intracellular ROS generation was precisely quantified using an advanced flow cytometer (Beckman Coulter, Brea, CA, USA).

### 2.6. Measurement of the Mitochondrial Membrane Potential (ΔΨm)

The JC-1 dye Detection Kit (Beyotime, Nantong, China) was used to determine the mitochondrial membrane potential (ΔΨm). Thus, the ratio of red to green fluorescence intensity, as quantified through flow cytometry, serves as a precise indicator of the mitochondrial membrane potential (ΔΨm) in each experimental group.

### 2.7. Determination of the ATP Content

The ATP content of HUVECs was measured with precision using an Enhanced ATP Assay Kit (S0027, Beyotime, Nantong, China). Following treatment, the HUVECs were meticulously harvested and standardized to a uniform cell count of 1 × 10^6^ per group. Adhering strictly to the manufacturer’s protocol, the quantification of ATP was performed by referencing the established standard curve, ensuring unparalleled accuracy and reliability.

### 2.8. Cell Transfection

The HUVECs were seeded in 6-well plates and transfected with the above plasmid (pcDNA3.1-Nrf2, pcDNA3.1, 500 ng) using Lipofectamine 2000 reagent (Invitrogen, Waltham, MA, USA) according to the manufacturer’s instructions.

### 2.9. qRT-PCR and Western Blotting Analysis

HUVECs with different treatments were collected. Total RNA was extracted using RNAiso (Takara, San Jose, CA, USA), and its quantity was measured by 260/280 UV spectrophotometry. Equal amounts of RNA (1.0 μg) were reverse-transcribed into cDNA using HiScript^®^ Q RT SuperMix (Vazyme, Nanjing, China). qPCR was performed using AceQ^®^ qPCR SYBR^®^ Green Master Mix (Vazyme) on an ABI StepONEPlus system. Relative gene expression was calculated using the 2^−ΔΔCt^ method [18]. Human GAPDH mRNA was used as an internal control, and the primers were listed in Table S1.

For western blotting, the treated HUVECs were collected and then lysed with radioimmunoprecipitation assay (RIPA, Vazyme Biotech Co., Ltd., Nanjing, China). For specific operations, we referred to the reported literature [39]. Antibodies included Nrf2 (16396-1-AP, Proteintech, Rosemont, IL, USA, 1:1000), Bax (50599-2-Ig, Proteintech, 1:1000), Bcl-2 (ET1603-11, HuaAn, 1:1000), PCNA (10205-2-AP, Proteintech, 1:1000), CCND1 (26939-1-AP, Proteintech, 1:1000), Caspase 3 (19677-1-AP, Proteintech, 1:1000), Caspase 8 (13423-1-AP, Proteintech, 1:1000), GAPDH (10494-1-AP, Proteintech, 1:10,000) and HSP90 (60318, Proteintech, 1:10,000), along with anti-rabbit IgG-HRP secondary antibody (1:10,000).

### 2.10. Data Analysis and Statistics

Statistical analyses were conducted, and figures were generated using GraphPad Prism software 8.0.1 (GraphPad Software, Inc., San Diego, CA, USA). A two-tailed Student’s *t*-test was employed to assess the significance between two groups. For the comparison of multiple components, a one-way ANOVA was used to calculate the *p* value among multiple groups. A *p*-value less than 0.05 was considered statistically significant.

## 3. Results

### 3.1. SFN Alleviates the Decrease in Cell Viability and Increases Inflammation of HUVECs upon High-Glucose Stimulation

**Figure 1:**
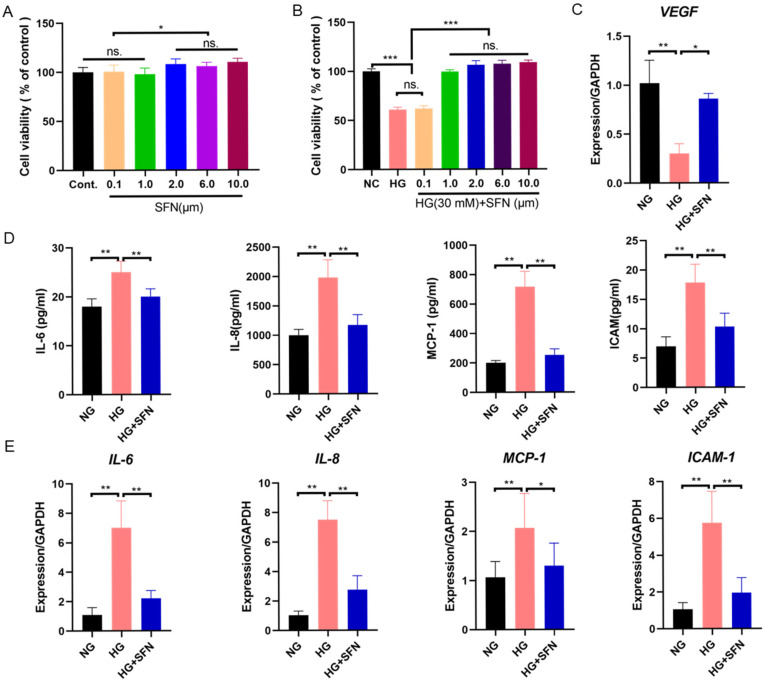
The effect of SFN and HG in HUVECs. (**A**). The effects of gradient concentration of Sulforaphane on HUVEC cell viability for 48 h. (**B**) The effects of combined treatment with SFN and HG on HUVEC cell viability for 48 h. (**C**) qRT-PCR analysis of the relative expression of VEGF mRNA levels in HUVECs. (**D**) The secretion and (**E**) mRNA expression of IL-6, IL-8, ICAM-1 and MCP-1. The data are expressed as means ± SD. Control: NG; Sulforaphane: SFN; High-glucose stimulation: HG; ns: No significant; * *p* < 0.05, ** *p* < 0.01, *** *p* < 0.001.

The high-glucose cultured HUVECs were used to construct the cell model of DFUs and to investigate the effects and mechanism of SFN under DFU conditions. Treatment with SFN alone at concentrations of 2.0, 6.0, and 10.0 μM slightly increased cell viability compared to the control and low-dose SFN (0.1 and 1.0 μM) (Figure 1A). In contrast, HG (30 mM) reduced cell viability, while co-treatment with SFN (1.0, 2.0, 6.0, and 10 μM) effectively mitigated the decrease in cell viability induced by HG treatment (Figure 1B). Meanwhile, VEGF exhibited a remarkable increase in mRNA levels following the administration of SFN treatment (Figure 1C). These results show that an appropriate amount of SFN can rescue HG-induced DFUs.

To determine the effects of high glucose (HG) and the protective role of sulforaphane (SFN) in diabetic foot ulcers (DFUs), we evaluated the inflammatory status of HUVECs after high-glucose-induced injury. ELISA and RT-qPCR were used to measure the impacts of HG and SFN on the secretion of IL-6, IL-8, ICAM-1, and MCP-1 proteins and mRNA expression. Results showed that HG increased the secretion and mRNA expression of these inflammatory factors; co-treatment with SFN (2 μM) effectively suppressed inflammation under DFU conditions (Figure 1D,E). Overall, HG reduced cell viability and increased inflammation in HUVECs, while SFN treatment alleviated the HG-induced damage in HUVECs.

### 3.2. SFN Alleviates the Cell Apoptosis in HUVECs upon High-Glucose Stimulation

**Figure 2:**
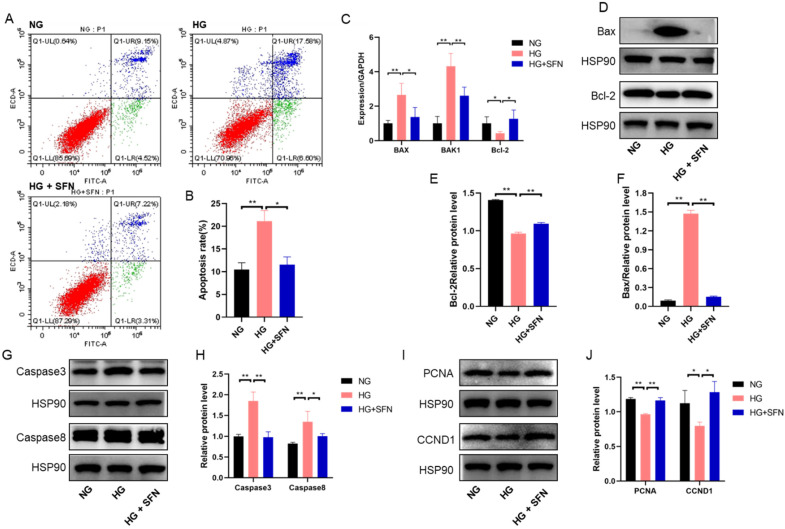
SFN treatment ameliorates the apoptosis of HUVECs induced by high-glucose stimulation. (**A**) Apoptosis of HUVECs was evaluated by the measurement of Annexin V using flow cytometry. Apoptotic cells were Annexin V-positive and PI-negative. The colors in different quadrants are different, representing different states. (**B**) The figure shows representative staining, and the numbers in the quadrants indicate the percentage of cells within the respective subpopulations. (**C**) qRT-PCR analysis of the relative expression of Bax, Bak and Bcl-2 mRNA levels in HUVECs (n = 3). (**D**–**F**) Western blotting analysis of the changes in the protein levels of related apoptosis factors (Bax and Bcl-2). (**G**,**H**) Western blotting analysis of the changes in the protein levels of related apoptosis factors (cleaved caspase-3 and caspase-8). (**I**,**J**) Western blotting analysis of the changes in the protein levels of functional genes linked to proliferation (PCNA and CCND1). Control: NG, Sulforaphane: SFN, High-glucose stimulation: HG. The data are expressed as means ± SD, * *p* < 0.05, ** *p* < 0.01.

To determine the protective effect of SFN in HUVECs under high-glucose stimulation, we analyzed cell apoptosis and related signaling pathways. High glucose (HG) significantly increased apoptosis compared to the control group, while SFN treatment reduced apoptosis to control levels (Figure 2A,B). HG also upregulated BAX and BAK1 mRNA levels and downregulated Bcl-2 mRNA levels; SFN reversed these effects (Figure 1C). Consistent with this, HG increased the protein levels of Bax, caspase-3, and caspase-8, while decreasing Bcl-2 levels. SFN co-treatment restored these proteins to near-control levels (Figure 2D–H). Additionally, HG reduced proliferation-related proteins PCNA and CCND1, which were restored by SFN co-treatment (Figure 2I,J). Together, these results indicate that SFN mitigates high-glucose-induced apoptosis and promotes HUVEC proliferation under DFU conditions.

### 3.3. SFN Alleviates Mitochondrial Dysfunction in HUVECs upon High-Glucose Stimulation

**Figure 3:**
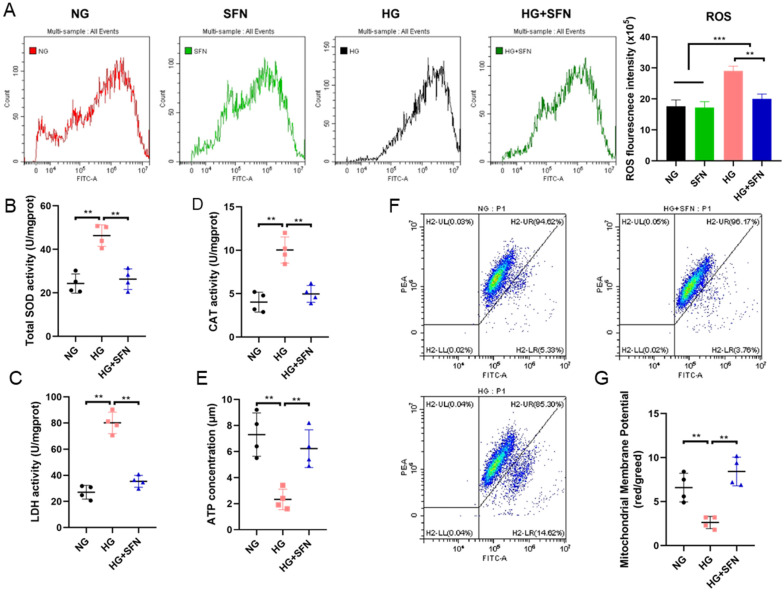
Effects of SFN on the mitochondrial function of HUVECs induced by high-glucose stimulation. (**A**) The ROS level of HUVECs in the different groups. (**B**–**E**) The SOD, CAT, HDL and ATP levels of HUVECs in different groups. Different colors represent different quadrants. (**F**) Mitochondrial membrane potential (ΔΨm) levels of the different groups. (**G**) The ratio of the red over green fluorescence intensity by flow cytometry represents the quantitative ΔΨm in each group. Control: NG; Sulforaphane: SFN; High-glucose stimulation: HG. The data are expressed as means ± SD. ** *p* < 0.01, *** *p* < 0.001.

A previous report indicated that high-glucose stimulation could induce oxidative stress in HUVECs [31]. We examined ROS levels in HUVECs after 48 h of high-glucose stimulation with or without SFN treatment. ROS signals significantly increased under high-glucose conditions, while SFN treatment reversed this effect, aligning ROS levels with those in the normal-glucose group (Figure 3A,B). In addition, superoxide dismutase (SOD), catalase (CAT) and lactate dehydrogenase (LDH) were measured in different treatments. The results indicated that high-glucose stimulation decreased the content of SOD, CAT and LDH, leading to the accumulation of ROS in HUVECs. However, the addition of SFN significantly increased the content of these antioxidant enzymes, thereby eliminating part of the accumulated ROS and alleviating oxidative stress in HUVECs upon high-glucose stimulation (Figure 3C,D).

To further investigate the protective effects of SFN on mitochondrial function in HUVECs under high-glucose conditions, we measured the ATP content and mitochondrial membrane potential (ΔΨm) following various treatments. High-glucose stimulation significantly decreased ATP content, an effect that was notably mitigated by co-treatment with SFN (Figure 3E). Furthermore, the results demonstrated that high-glucose stimulation significantly increased green fluorescence intensity (Figure 3F,G, HG) compared to the normal glucose (NG) group (Figure 3F,G, NG). However, the addition of SFN markedly reduced green fluorescence intensity (Figure 3F,G, HG + SFN) relative to the HG group. Additionally, alterations in ΔΨm were quantified by calculating the ratio of red fluorescence intensity to green fluorescence intensity. High-glucose exposure led to a significant decrease in this ratio compared to the control group, whereas co-treatment with SFN effectively attenuated this decline (Figure 3G). Collectively, these findings indicate that SFN ameliorates mitochondrial dysfunction in HUVECs induced by high-glucose conditions.

### 3.4. SFN Mediates Nrf2 Expression to Inhibit HG-Induced Oxidative Stress Damage in HUVECs

**Figure 4:**
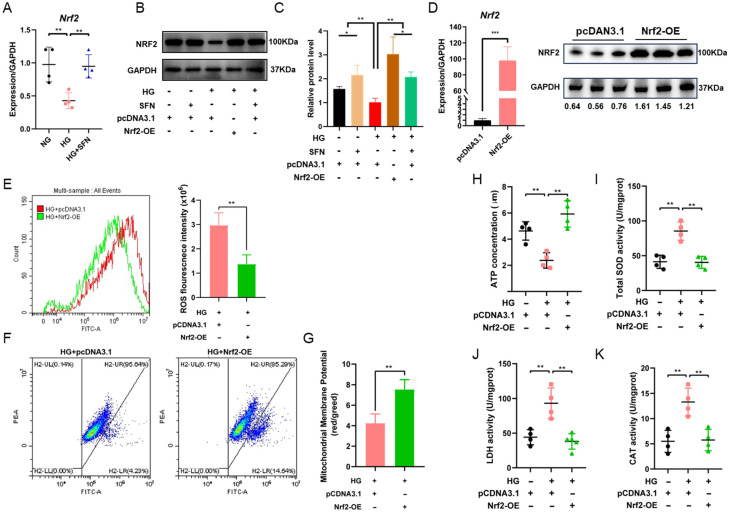
SFN mediates Nrf2 expression to inhibit HG-induced oxidative stress damage in HUVECs. (**A**–**C**) The mRNA and protein expression of Nrf2 under high-glucose stimulation and SFN. (**D**) The mRNA and protein expression of Nrf2 in HUVECs after cell transfection. (**E**) The ROS level of HUVECs in the different groups after Nrf2 overexpression. (**F**,**G**) Mitochondrial membrane potential (ΔΨm) levels of the different groups after Nrf2 overexpression. (**H**–**K**) The SOD, CAT, LDH and ATP levels of HUVECs in different groups after Nrf2 overexpression. Sulforaphane: SFN; High-glucose stimulation: HG. The data are expressed as means ± SD, * p < 0.05, ** p < 0.01, *** *p* < 0.001.

Initially, we explored the impact of SFN supplementation on Nrf2 expression, revealing that SFN markedly upregulates NRF2 levels (Figure 4A). Subsequently, the expression level of Nrf2 was detected under high-sugar stimulation conditions. The results showed that high sugar significantly downregulated the mRNA and protein expression of Nrf2, while combined treatment with SFN could effectively reverse this inhibitory effect and significantly enhance the expression of Nrf2 at both transcriptional and translational levels (Figure 4B,C). These results showed that SFN significantly elevated Nrf2 levels under high-glucose stimulation. Therefore, we speculated that SFN alleviates DFU damage induced by high-glucose stimulation through or depending on NRF2 activation. To validate the hypothesis and examine the relationship between Nrf2 activation and DFU conditions, the genetic manipulation was performed to overexpress Nrf2 in HUVECs (Figure 4D). Next, the relative markers of oxidative stress were detected after overexpress Nrf2 under DFU conditions. Notably, Nrf2 overexpression decreased the accumulation of ROS (Figure 4E) and alleviated the decline in ΔΨm under high-glucose stimulation (Figure 4F,G). In addition, Nrf2 overexpression effectively reversed the decline of ATP, SOD, CAT and LDH content in HUVECs induced by high-glucose stimulation (Figure 4H–K). These results indicated that SFN mediates Nrf2 expression to inhibit HG-induced oxidative stress damage in HUVECs.

### 3.5. SFN Mediates Nrf2 Expression to Restore Cell Viability and Reduce Inflammation in HUVECs Under High-Glucose Conditions

**Figure 5:**
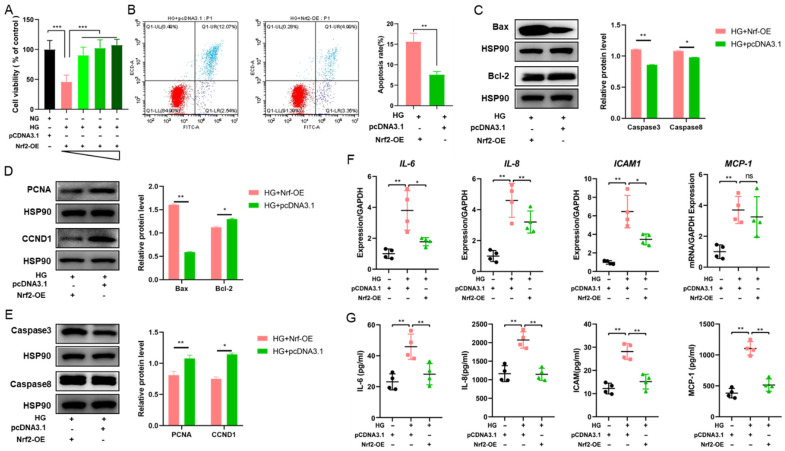
SFN mediates Nrf2 expression to restore cell viability and reduce inflammation in HUVECs under high-glucose stimulation. (**A**) The cell viability was analyzed by CCK-8 in HUVECs transfected with increasing doses of Nrf2 plasmids (0, 0.5, 1.0 and 2 μg). (**B**) The apoptosis analysis of HUVECs after Nrf2 overexpression. (**C**–**E**) Western blotting analysis of the changes in the protein levels of related apoptosis factors (Bax, Bcl-2, cleaved caspase-3, caspase-8, PCNA and CCND1) after Nrf2 overexpression. (**F**,**G**) The secretion and mRNA expression of IL-6, IL-8, ICAM-1 and MCP-1 after Nrf2 overexpression. Sulforaphane: SFN; High-glucose stimulation: HG. The data are expressed as means ± SD, * *p* < 0.05 and ** *p* < 0.01, *** *p* < 0.001.

To further verify the protective effect of SFN under high-glucose stimulation by activating NFR2, we examined the alterations in cell viability and inflammatory response induced by hyperglycemic stimulation after NRF2 overexpression. Hence, we detected the cell viability of HUVECs under high-glucose stimulation with transfected Nrf2 plasmid (0, 0.5, 1.0 and 2.0 μg) and found that Nrf2 overexpression effectively reversed the decline in cell viability induced by high-glucose stimulation (Figure 5A). Nrf2 overexpression inhibited the apoptosis of HUVECs induced by high-glucose stimulation (Figure 5B). In addition, the levels of several related apoptosis factors were detected; the results indicated that the overexpression of Nrf2 reversed the increase in Bax, caspase 3 and caspase 8 and the decrease in Bcl-2, PCNA and CCND1 (Figure 5C–E), which was in line with the results of the treatment with SFN (Figure 2D–F). The results indicated that SFN mediates Nrf2 expression and alleviates the decline in cell viability in HUVECs under DFU conditions.

Moreover, the ELSIA and RT-qPCR were performed to detect the mRNA expression and secretion levels of IL-6, IL-8, ICAM-1 and MCP-1 under high-glucose conditions. The results indicated that the mRNA expression and secretion of IL-6, IL-8 and ICAM-1 were enhanced by high glucose, whereas Nrf2 overexpression attenuated these HG-induced increases (Figure 5F,G). Among them, the mRNA expression level of MCP-1 did not show a significant change after Nrf2 overexpression; however, the secretion of MCP-1 was reduced with the transfection of Nrf2 overexpression under high-glucose conditions (Figure 5F,G). The results indicated that SFN mediates Nrf2 expression and alleviates inflammation in HUVECs under high-glucose stimulation.

## 4. Discussion

Slow-progressing but persistent DFUs are the leading cause of non-traumatic lower limb amputations and a significant public health burden in developing countries [4]. Therefore, in-depth research on the pathogenesis of DFU and the search for effective therapeutic targets and drugs are of great significance for clinical treatment and even reversal of DFU [7]. DFU is mainly caused by persistent inflammation resulting from a deficiency in tissue repair and reaction in glycosuria disease; thus, effectively alleviating inflammation and reducing the wound stress response is an important measure in surface treatment of DFU wounds. A large number of research results show that one of the major factors causing the occurrence and development of diabetic foot disease is the oxidative stress caused by high blood sugar, which plays an important role in the pathogenesis of diabetic foot ulcers [40]. In our study, SFN, as a prominent antioxidant, was applied to DFU cell models, and it was found that it could effectively reduce oxidative stress induced under high-glucose stimulation, as shown by decreased ROS accumulation and increased levels of SOD, CAT, LDH and ATP. At the molecular level, sulforaphane modulates cellular homeostasis via the activation of the transcription factor Nrf2, while, which is well-known to be involved in mitochondrial regulation through different mechanisms [41]. In the present study, numerous ROS were generated and accumulated in the cytoplasm and mitochondria under high-glucose stimulation, leading to the dysfunction of mitochondria, which aligns with the function of Nrf2 [42]. Therefore, the study indicated that SFN modulated mitochondrial function and reduced oxidative stress under high-glucose stimulation through the activation of Nrf2, suggesting that SFN can be used as a potential target drug for DFU therapy from the perspective of antioxidants.

SFN is a natural bioactive compound found in cruciferous vegetables such as broccoli, formed by the hydrolysis of its precursor glucoraphanin by the enzyme myrosinase. Its core mechanism involves activating the intracellular Nrf2 signaling pathway [26], which induces the expression of various Phase II detoxifying enzymes and antioxidant proteins [16], thereby exerting potent antioxidant [22,23], anti-inflammatory [26] and cytoprotective effects [31]. Notably, dietary sulforaphane (SFN) has been shown to cross the placental barrier, reach the fetal circulation, and activate both Nrf2 and its downstream transcriptional targets in the fetus [39]. A key question remains: how does SFN mediate Nrf2 activation? Mechanistic studies reveal that SFN acts as an electrophilic compound that employs a calcium-dependent, yet mTOR-independent, pathway. This unique signaling mechanism elevates intracellular reactive oxygen species (ROS) to moderate levels, subsequently promoting nuclear translocation of the transcription factor TFEB [43]. As Nrf2 is a recognized downstream target of TFEB [43], this pathway clearly delineates why Nrf2 emerges as a principal molecular target of SFN. This rapid activation of TFEB by SFN involves its fast kinetic properties, specifically through the rapid release of calcium ions, which in turn drives TFEB [44]. Therefore, there is still much to be explored regarding the mechanism by which SFN activates Nrf2.

In addition, high-glucose stimulation could cause a significant inflammatory response in HUVECs, which is very similar to the clinical phenotype of DFU wounds. Reducing the inflammatory state of the surface would help the healing of the ulcer surface [45]. Many studies have tried to use anti-inflammatory drugs to treat the inflammatory response of DFU wounds in order to promote wound healing, such as Bletilla striata polysaccharide promoting DFU wound healing via inhibition of hyperactivation of the inflammatory corpuscles of macrophage cell NLRP3 in the ulcer area [46]. Dracorhodin perchlorate inhibits the inflammatory response of wounds and weakens the inflammatory infiltration of wounds by inhibiting the secretion of inflammatory factors such as IL-lα and TNF-a [47]. Zizhu Ointment promotes the healing of diabetic foot ulcers by reducing the inflammation level of diabetic foot ulcers infected by Staphylococcus aureus and inhibiting the inflammatory response to accelerate the healing of diabetic foot ulcers. This study shows that it is reasonable and wise to explore targeted drugs for DFU therapy from the perspective of anti-inflammation. In this study, SFN could alleviate cell damage in the DFU cell model by reducing the mRNA expression and secretion of inflammatory factors (including IL-6, IL-8, ICAM-1, and MCP-1), which proved that SFN had a significant anti-inflammatory effect on DFU conditions. Combined with NRF2, a key gene targeted by SFN [43], we found that the overexpression of NRF2 could inhibit the mRNA expression and secretion of IL-6, IL-8, ICAM-1, and MCP-1, which is in line with the addition of SFN. These results indicate that SFN could rely on the activation of Nrf2 to inhibit the inflammatory response, reducing cell damage in the DFU cell model. In this study, HUVECs treated with high glucose were used as an in vitro model to simulate changes in endothelial function in the diabetic environment. However, this model still has some limitations. First, it is a single-cell system and does not cover the multicellular interactions of keratinocytes, fibroblasts and immune cells. Second, the important microenvironmental factors of diabetic wounds, such as hypoxia, inflammation, and microbial infection, were not included. Therefore, this model is mainly used to explore the direct effects of high glucose on endothelial cells, and it is difficult to fully reflect the complex pathological process of diabetic wounds. Future studies with more complex models are needed to verify the results.

## 5. Conclusions

**Figure 6:**
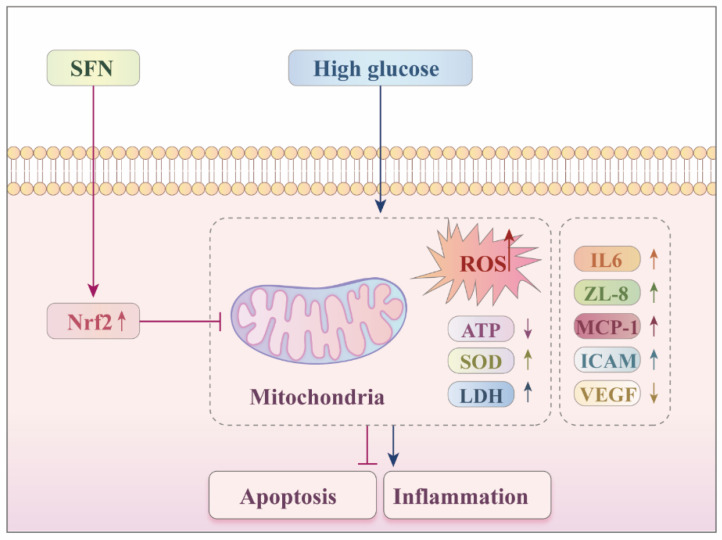
The potential molecular mechanism of SFN on DFUs. SFN may activate the expression of Nrf2, which regulates the maintenance of the mitochondrial homeostasis and the inflammatory response. The upward arrow indicates an upregulation; the downward arrow indicates a downregulation; the T-shaped (blunt-ended) line represents an inhibitory effect.

Taken together, this study reported that under diabetic ulcer conditions, SFN could activate the expression of NRF2, effectively alleviate cell damage in HUVECs upon high-glucose stimulation, including cell viability, mitochondrial function and inflammatory response. These findings provide a mechanistic rationale for further evaluating SFN as a potential therapeutic candidate for DFU, particularly in in vivo diabetic wound models with optimized delivery/formulation (Figure 6). However, for the transcription factor NRF2, the regulatory network in its activated state is more complex, which is an important research direction for us to explore the application of SFN in DFU therapy in the future.

## 6. Limitations of the Study

In this study, we preliminarily revealed the protective role of sulforaphane (SFN) in a high-glucose-induced diabetic foot ulcer (DFU) cell model and suggested that its mechanism may involve the activation of the Nrf2 pathway. However, the specific regulatory network requires further in-depth investigation. Although SFN demonstrated alleviating and reversing effects on high-glucose-induced damage through Nrf2 in cellular models, a comprehensive evaluation of its pharmacological properties and clinical potential still necessitates the establishment of robust animal models to validate its therapeutic efficacy in diabetic foot treatment and wound healing.

## Data Availability

The original contributions presented in this study are included in the article. Further inquiries can be directed to the corresponding authors.

## Supplementary Materials

The following supporting information can be downloaded at: https://www.mdpi.com/article/10.3390/biomedicines14050997/s1, Table S1: Primers used for quantitative RT-PCR.

## Abbreviations

The following abbreviations are used in this manuscript:

DM	Diabetes mellitus
DFUs	Diabetic foot ulcers
HUVECs	Human umbilical vein endothelial cells
