# Supplementary material for: Sulforaphane Ameliorates High-Glucose-Induced Damage in a Diabetic Foot Ulcer Cell Model by Activating the Nrf2 Pathway to Improve Mitochondrial Function and Suppress Inflammation
Source: Biomedicines. 2026 Apr 27;14(5):997. doi: 10.3390/biomedicines14050997 (PMC13203912; doi:10.3390/biomedicines14050997)
Supplement: Supplementary file 1 [file biomedicines-14-00997-s001.zip › biomedicines-4189352-supplementary.pdf]

Supplementary Table S1. Primers used for quantitative RT-PCR.

Name	Sequence (5′ - 3′)
Nrf2	Forward: CACATCCAGTCAGAAACCAGTGG<br>Reverse: GGAATGTCTGC GCCAAAAGCTG
IL-6	Forward: CCGAAGCAAACATCACATTCA<br>Reverse: GGTCTAAAGGCTCCGGGCT
ICAM-1	Forward: GTAGCCCACGTCGTAGCAAA<br>Reverse: ACAAGGTACAACCCATCGGC
MCP-1	Forward: GAGGACATGAGCACCTTCTTT<br>Reverse: GCCTGTAGTGCAGTTGTCTAA
IL-8	Forward: CCCTTTGCTATGGTGTCCTTTC<br>Reverse: AGGATCTCCCTGGTTTCTCTTC
Bax	Forward: GAACGTCGAAAAGAAAAGTCTCG<br>Reverse: CCTTATCAAGATGCGAACTCACA
BAK	Forward: GTGTGTGGAATTTGATGGG<br>Reverse: CAGCTTTGTCCAACAGAGG
Bcl-2	Forward: GGGGCAAGGTGGAACAGTTAT<br>Reverse: CCGCTTGGAGTGTATCAGTCA
GAPDH	Forward: TGACATCAAGAAGGTGGTGA<br>Reverse: TCCACCACCCTGTTGCTGTA
